# Colorimetric Food Freshness Indicators for Intelligent Packaging: Progress, Shortcomings, and Promising Solutions

**DOI:** 10.3390/foods14162813

**Published:** 2025-08-14

**Authors:** Xiaodong Zhai, Yuhong Xue, Yue Sun, Xingdan Ma, Wanwan Ban, Gobinath Marappan, Haroon Elrasheid Tahir, Xiaowei Huang, Kunlong Wu, Zhilong Chen, Wenwu Zou, Biao Liu, Liang Zhang, Zhikun Yang, Jaroslav Katona

**Affiliations:** 1 School of Food and Biological Engineering, Jiangsu University, Zhenjiang 212013, China; zhai_xiaodong@ujs.edu.cn (X.Z.); xue_yuhong@163.com (Y.X.); 1000006728@ujs.edu.cn (Y.S.); xingdanma@163.com (X.M.); 13275356807@163.com (W.B.); gobinathnst@gmail.com (G.M.); haroona28@yahoo.com (H.E.T.); 2 Institute of Modern Agriculture and Health Care Industry, Wencheng, Wenzhou 325300, China; zhilongchen@sina.com (Z.C.); 13968830330@163.com (W.Z.); liubiao17@163.com (B.L.); liangzhang_xj@163.com (L.Z.); 3 School of Tourism and Culinary Science, Yangzhou University, Yangzhou 225127, China; yangzhikun@yzu.edu.cn; 4 Faculty of Technology Novi Sad, University of Novi Sad, Bul. cara Lazara 1, 21000 Novi Sad, Serbia; jaroslav.katona@uns.ac.rs

**Keywords:** colorimetric, freshness indicators, intelligent packaging, food freshness, food safety

## Abstract

The colorimetric food freshness indicator (CFFI) is a promising technology in intelligent food packaging, offering the capability for real-time monitoring of food freshness through colorimetric changes. This technology holds significant promise in mitigating food waste and enhancing transparency across the supply chain. This paper provides a comprehensive review of the classification system for the CFFI, encompassing colorimetric films and sensor arrays. It explores their applications across key perishable food categories, including meats, seafoods, fruits, and vegetables. Furthermore, this paper offers an in-depth analysis of three critical challenges currently hindering technological advancement: safety concerns, stability issues, and limitations in sensitivity and selectivity. In addressing these challenges, this paper proposes forward-looking solutions and outlines potential research directions aimed at overcoming these bottlenecks, thereby fostering substantial progress in the development of this field.

## 1. Introduction

Intelligent food packaging (IFP) represents a significant breakthrough in food technology, integrating advanced smart systems into conventional packaging to actively monitor, communicate, and enhance food quality, safety, and shelf life [1,2]. In contrast to traditional packaging, which serves primarily as a passive barrier, IFP actively interacts with the food product or its surrounding environment through embedded sensors (e.g., gas or pH sensors), indicators (e.g., colorimetric or microbial), and data carriers (e.g., quick response codes or radio-frequency identification tags) [3,4]. These systems enable real-time, on-package monitoring of critical parameters such as freshness biomarkers (e.g., volatile amines), temperature history (via time-temperature integrators), microbial activity, and headspace gas composition (e.g., O_2_/CO_2_ levels), thereby empowering stakeholders across the supply chain to make data-driven decisions [5]. Intelligent food packaging technology is particularly important for perishable foods such as meat, dairy, seafood, and fresh produce, where improper storage or spoilage results in substantial waste—approximately 1.3 billion tons annually [6]. By incorporating smart labels, time–temperature indicators (TTI), radio-frequency identification tags (RFID), and biosensors, IFP not only mitigates waste but also improves regulatory compliance, reduces economic losses, and strengthens consumer confidence through enhanced transparency [7,8,9].

The CFFI generally refers to those indicators that could exhibit color changes associated with food freshness [10]. Compared with RFID and electronic TTI, the power-free CFFI is more convenient to use and typically offers a lower cost. The CFFI can be categorized based on its functional principles, including colorimetric films, colorimetric sensor arrays, and time–temperature indicators [11]. A colorimetric film typically consists of one or more dyes immobilized on a solid film substrate [12,13,14]. These films exhibit direct color changes that correlate well with food freshness parameters. In contrast, colorimetric sensor arrays comprise multiple discrete dyes, each immobilized on a separate solid substrate [15,16]. Unlike colorimetric films that rely on single-point colorimetric responses requiring minimal data processing, sensor arrays generate multi-point response patterns that necessitate multivariate regression analysis for accurate interpretation [17,18]. Colorimetric time–temperature indicators function through either physical mechanisms (e.g., controlled dye diffusion) or chemical reactions (e.g., hydrolysis or polymerization), with their color transition kinetics being precisely calibrated to reflect cumulative time–temperature exposure [19,20,21,22].

**Figure 1:**
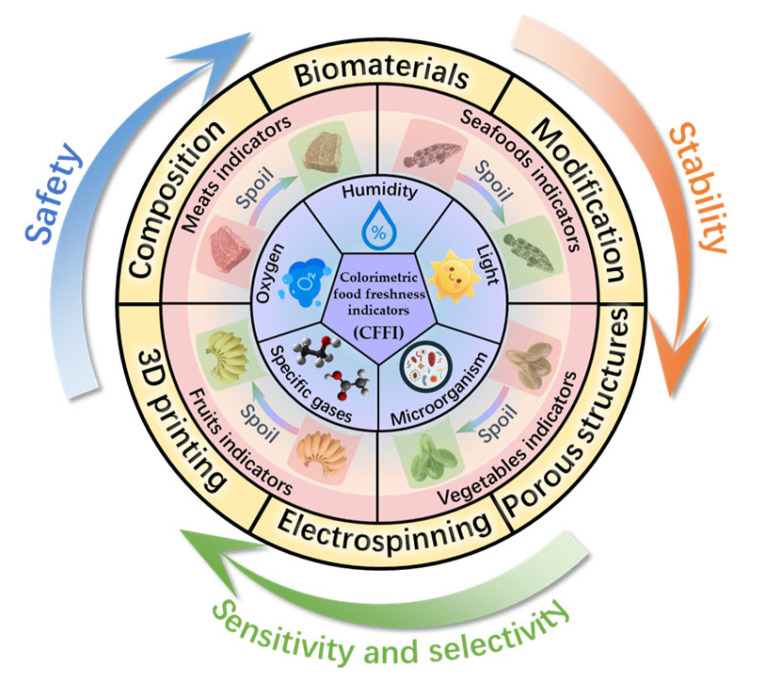
Schematic representation of progress, shortcomings, and promising solutions of CFFI.

In recent years, systematic research has been conducted to explore advancements in various types of CFFIs for intelligent food packaging applications, with a particular focus on their classification schemes, material compositions, and functional mechanisms [2,4,23,24,25,26]. While existing review articles have predominantly highlighted the advantages of CFFI technologies, there remains a notable gap in the critical assessment of their current limitations. To bridge this gap and offer a more comprehensive assessment, the present study places particular emphasis on identifying and analyzing the prevailing technical challenges associated with CFFI systems. Through this critical examination, we aim to provide researchers with targeted directions for future development, ultimately facilitating the advancement of this field by addressing these identified shortcomings. Figure 1 presents a schematic representation illustrating the progress, shortcomings, and promising solutions of CFFIs.

## 2. CFFI Types Based on Application Object

### 2.1. Meats and Seafoods Freshness Indicators

**Figure 2:**
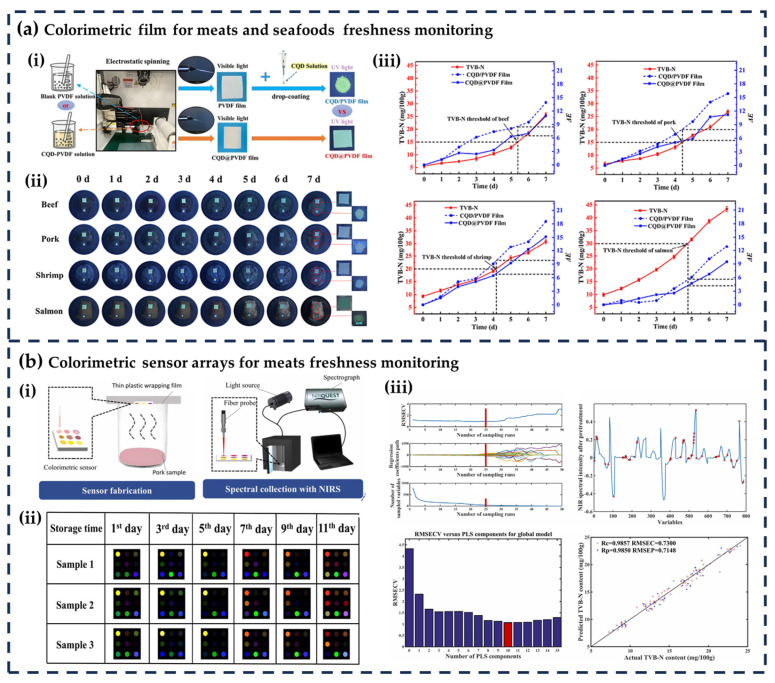
(**a**) Colorimetric film for meats and seafoods freshness monitoring (**i**) fabrication of the film, (**ii**) color change of the film, and (**iii**) TVB-N and ΔE values when monitoring the freshness of beef, pork, salmon, and shrimp [33]; (**b**) colorimetric sensor arrays for meats freshness monitoring (**i**) fabrication of the sensor arrays, (**ii**) color change of the sensor arrays as the storage time of pork increases, and (**iii**) model establishment for TVB-N [48]. All the images are reproduced with permission from Elsevier.

Meats and seafoods are highly perishable due to their high moisture content, protein richness, and susceptibility to microbial spoilage and enzymatic degradation [27,28,29]. Colorimetric indicators have emerged as effective tools for real-time freshness monitoring by detecting spoilage metabolites such as volatile amines (TVB-N), hydrogen sulfide (H_2_S), biogenic amines (histamine, putrescine), and pH changes. Table 1 presents the application of CFFIs in the freshness monitoring of meats and seafoods, while Figure 2a,b illustrate examples of the application of different indicators in meats and seafoods.

**Table 1:** Application of CFFI for meats and seafoods freshness monitoring.

Indicator Type	Indicator Components	Preparation Method	Food Type	Target Compound	Color Change	Ref.
Colorimetric films	Methyl red, bromocresol green	Immobilize	Beef	TVB-N	Red to yellow yellow to purple	[30]
	Methyl red, bromocresol blue	Casting-drying	Chicken breast	VOCs (e.g., CO_2_)	Green to orange yellow	[31]
	Anthraquinone, azo chromophore	Screen printing	Crab cooked	VOCs (e.g., NH_3_, HCl)	Green to purple to red	[32]
	Dual-emission carbon quantum dots	Electrospinning	Beef, pork and shrimp	TVB-N	Yellow green to blue	[33]
	Silicon quantum dots and silver nanoclusters	Added onto a PVDF film	Beef	VOCs (e.g., H_2_S and CH_3_SH)	Purplish to cyan	[34]
	Ag nanoparticles	Casting	Chicken breast and silver carp	VOCs (e.g., H_2_S)	Yellow to colorless	[35]
	Curcumin	Electrochemical printing	Freshwater shrimp	pH	Yellow to red	[36]
	Curcumin	Melting extrusion	Beef and silver carp	TVB-N	Light yellow to light brown	[37]
Colorimetric films	Alizarin	Dip-coating	Fish fillet	VOCs (e.g., ammonia)	Yellow to purple	[38]
	Cyanidin, alizarin	Casting-drying	Pork	VOCs (e.g., ammonia)	Red to blue/black	[12]
	Alizarin	Casting-drying	Beef	VOCs (e.g., ammonia)	Yellow to purple	[39]
	Alizarin	Electrospinning	Pork	TVB-N	Yellow to purple	[40]
	Red radish anthocyanins	Electrochemical writing	Fish	TVB-N	Orange/red to green to yellow/green	[41]
	Purple sweet potato anthocyanins	3D-print	Beef and salmon	VOCs (e.g., volatile amines)	Red to purple	[42]
	Red cabbage anthocyanins	Dip-coating	Pork, chicken, salmon, and shrimp	TVB-N	Pink to green	[14]
	Mulberry anthocyanins	Electrochemical writing	Crucian	TVB-N	Pink to light green to yellow/green	[43]
Colorimetric sensor arrays	16 chemically sensitive compounds	Drop-casting	Fish	VOCs (e.g., volatile amines)	Different dyes with different color changes	[44]
	8 pH indicators, 8 porphyrins	Drop-casting	*Yao*-meat	VOCs (e.g., trimethylamine)	Different dyes with different color changes	[15]
	Nile red, zinc tetraphenylporphyrin and methyl red	Drop-casting	Chicken breast	pH and VOCs (e.g., ethanol, methanol, toluene)	Different dyes with different color changes	[45]
	9 porphyrins or metalloporphyrins, bromocreslo green, bromocresol purple and neutral red	Drop-casting	Chicken	VOCs (e.g., ethanol, methanol, toluene)	Different dyes with different color changes	[46]
Colorimetric sensor arrays	6 pH indicators, 9 porphyrin compounds, and 1 metal-phthalocyanine	Drop-casting	Fish	VOCs (e.g., ethanol, acetic acid, trimethylamine)	Different dyes with different color changes	[47]
	6 porphyrins and 3 hydrophobic pH indicators	Drop-casting	Pork	VOCs (e.g., acetaldehyde, H_2_S, and ammonia)	Different dyes with different color changes	[48]
	12 porphyrin materials and 8 pH indicators	Drop-casting	Snakehead fillets	TVB-N	Different dyes with different color changes	[49]
	3 pH markers and 9 metalloporphyrins	Drop-casting	Chicken meat	TVB-N	Different dyes with different color changes	[50]
	Pyridylazo and porphyrin indicators	Drop-casting	Fish	Pb, Cd and Hg	Different dyes with different color changes	[51]
	6 metalloporphyrins and 1 protoporphyrin	Drop-casting	Mackerel	VOCs (e.g., trimethylamine)	Different dyes with different color changes	[52]
	4 pyridine azo compounds and 4 porphyrin compounds	Drop-casting	Large yellow croakers	Lead	Different dyes with different color changes	[53]

From the applications summarized in Table 1, it is evident that the primary target compounds for monitoring the freshness of meats and seafoods are volatile basic nitrogen (TVB-N), followed by volatile organic compounds (VOCs). These metabolites are key markers reflecting microbial spoilage and enzymatic degradation in such products. In terms of indicator components, many recent studies have used natural pigments instead of chemically synthetic indicators, considering factors such as safety and environmental friendliness. Anthocyanins, curcumin, and alizarinfrom are widely used due to their reliable color changes in detecting TVB-N, ammonia, and pH. However, synthetic compounds like methyl red, bromocresol green/blue, and porphyrins are equally important, particularly in colorimetric sensor arrays, where their combination enables multi-target detection of VOCs and biogenic amines.

Notably, colorimetric sensor arrays, composed of multiple sensitive compounds (e.g., porphyrins, pH indicators), exhibit higher sensitivity compared to single-component colorimetric films. This is attributed to their ability to respond to a broader range of spoilage metabolites, providing comprehensive freshness information. Among preparation methods, casting–drying and electrospinning are most prevalent: casting–drying offers simplicity and scalability, while electrospinning enhances sensitivity by creating porous structures with large surface areas. Most indicators reacted with volatile metabolites of foods without direct contact with foods, thus avoiding adverse effects on foods’ physical properties or safety.

### 2.2. Fruits and Vegetables Freshness Indicators

**Figure 3:**
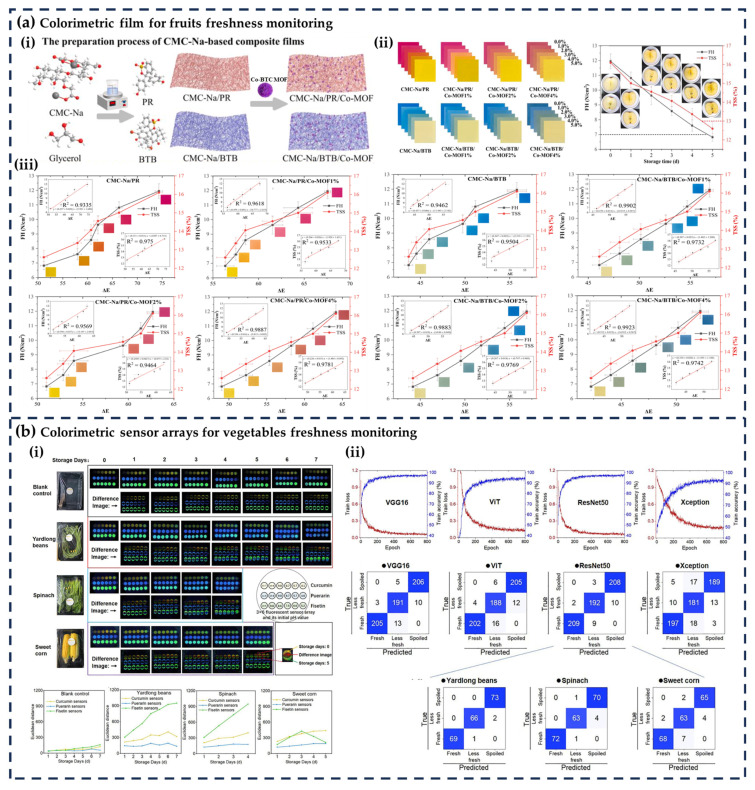
(**a**) colorimetric film for fruits freshness monitoring (**i**) fabrication of the film, (**ii**) color change of the films with CO_2_ concentration and FH, TSS changes of fresh-cut during storage, and (**iii**) correlation of films’ Δ*E* with FH and TSS of fresh-cut apples [57]; (**b**) colorimetric sensor arrays for vegetables freshness monitoring (**i**) color change of the sensor arrays as the storage time of three vegetables and (**ii**) DCNN model establishment for freshness prediction of three vegetables [80]. All the images are reproduced with permission from Elsevier.

Fruits and vegetables are highly perishable due to their high water content, respiratory activity, and susceptibility to microbial growth and enzymatic processes. Colorimetric indicators have proven effective in monitoring freshness by detecting spoilage-related compounds such as ethylene, organic acids, and pH changes [54]. Colorimetric indicators provide a reliable method for real-time assessment of produce quality and ripeness, facilitating optimal storage and timely consumption. Table 2 presents the application of CFFIs in the freshness monitoring of fruits and vegetables, while Figure 3a,b illustrate examples of the application of different indicators in fruits and vegetables.

**Table 2:** Application of CFFI for fruits and vegetables freshness monitoring.

Indicator Type	Indicator Components	Preparation Method	Food Type	Target Compound	Color Change	Ref.
Colorimetric films	Methyl red, bromocresol blue	Casting–drying	Fresh cut green pepper	VOCs (e.g., CO_2_)	Yellow green to orange	[55]
	Methyl red and bromothymol blue	Casting–drying	Green bell pepper and greengrocery	VOCs (e.g., CO_2_)	Orange to red	[56]
	Phenol red, bromothymol blue	Casting–drying	Fresh-cut apple	VOCs (e.g., CO_2_)	Purple red to yellow Dark blue to yellow	[57]
	Ammonium molybdate	Casting–drying	Avocados	VOCs (e.g., ethylene)	Yellow to greenish yellow	[58]
	Ammonium molybdate, palladium sulfate	Immerse	Apple	VOCs (e.g., ethylene)	Light yellow to dark blue	[59]
	Red phenanthroline	Immerse	Kiwi fruit	VOCs (e.g., ethylene)	Beige to dark brown	[60]
	Potassium dichromate and sulfuric acid	Immerse	Mango fermented	VOCs (e.g., ethylene)	Yellow to blue	[61]
	Poly (Ethylene Glycol) bis(3-aminopropyl) terminated (amine-PEG), methyl red	Casting–drying	Kiwi fruit	VOCs (e.g., ethylene)	Yellow to orange to red	[62]
	Methyl red, methyl red sodium salt	Printing inks	Apple	VOCs (e.g., ethylene)	Yellow to orange to red	[63]
Colorimetric films	Bromophenol blue	Immobilize	Guavas	VOCs (e.g., organic acids)	Blue to green	[64]
	Brazilian plant extract dye	Casting–drying	Banana	pH	Yellow to red	[65]
	Bromothymol blue, methyl red	Casting–drying	Fresh-cut durian	pH	Red to orange	[66]
	Purple sweet potato anthocyanins, Silver nanoparticles	Casting–drying	Strawberry	pH	Purple to yellow/purple	[67]
	Red cabbage anthocyanins extract	Casting–drying	Mushroom	pH	Reddish brown to light brown	[68]
	Anthocyanins	Casting–drying	Grape	pH	Yellow/brown to purple	[69]
	Black wolfberry and red cabbage anthocyanins	Casting–drying	Fresh-cut pineapple	pH	Purple to red	[70]
	Purple sweet potato anthocyanins	Casting–drying	*Flammulina velutipes* mushroom	pH	Green to purplish gray to yellow	[71]
	Purple sweet potato anthocyanins	Immerse	White oyster mushrooms	pH	Dark purple to light purple to green	[72]
Colorimetric sensor arrays	6 porphyrins and 6 pH indicators	Drop-casting	Potato	VOCs (e.g.,2,3-Butanediol and ethyl ester)	Different dyes with different color changes	[73]
	20 color-sensitive materials	Applied using a capillary	Soybeans	VOCs (e.g., acetic acid)	Different dyes with different color changes	[74]
	4 pH indicators, 2 developed dyes, and 9 porphyrin compounds	Drop-casting	Mango	VOCs (e.g., ethylene)	Different dyes with different color changes	[75]
	5 phages	Drop-casting	Banana	VOCs (e.g., 2-pentanone and 3-methyl-1-butanol)	Different dyes with different color changes	[76]
Colorimetric sensor arrays	Bromocresol purple, methyl orange, thymol blue, and bromocresol green	Dip	Bananas, apples, and pears	VOCs (e.g., acetaldehyde, propionaldehyde and acetone)	Different dyes with different color changes	[77]
	8 dye/MOF composites and 2 Pd^2+^/dye/MOF composites	Deposit	Banana	VOCs (e.g., ethylene, ethanol, and ethyl acetate)	Different dyes with different color changes	[78]
	15 sensing materials	Print	Garlic, green pepper, and nectarine	VOCs (e.g., sulfur-based volatiles)	Different dyes with different color changes	[79]
	Curcumin, puerarin, and fisetin	Drop-casting	Yardlong beans, spinach, and sweet corn	VOCs (e.g., indole, nitrogen-containing volatiles and acetic acid)	Different dyes with different color changes	[80]

Table 2 highlights that the freshness monitoring of fruits and vegetables primarily targets carbon dioxide (CO_2_), ethylene, aldehydes, and pH changes, which are the key indicators of respiratory activity, ripening, and microbial spoilage. A prominent trend is the widespread use of plant-derived anthocyanins (e.g., from purple sweet potato, red cabbage, and black wolfberry), which offer natural, safe, and pH-sensitive color transitions, aligning with the growing demand for eco-friendly food packaging. Additionally, pH indicators (e.g., methyl red, bromothymol blue) and ethylene-sensitive compounds (e.g., ammonium molybdate) are highly effective, with consistent responses to CO_2_ and ethylene. Fruits and vegetables with the most extensive studies include apples, bananas, mangoes, green peppers, and mushrooms, likely due to their high perishability and commercial importance. Casting–drying is the most widely used preparation method for colorimetric films, valued for its simplicity and suitability for large-scale production, while drop-casting and printing are favored for sensor arrays to immobilize multiple sensitive materials.

## 3. Current Main Shortcoming for CFFIs

Although there has been growing research on CFFIs, the majority of these indicators continue to face significant challenges in practical applications. These challenges are primarily due to limitations in safety, stability, and sensitivity. Notably, a successful freshness indicator must simultaneously satisfy the requirements of all three aspects mentioned above. This dual requirement scenario, including the individual inadequacies and the need for concurrent fulfillment, substantially complicates the development process of such indicators, thereby posing a great challenge for researchers in this field.

### 3.1. Safety

The safety of freshness indicators ranks as the foremost concern that demands meticulous consideration. Given that these indicators are an integral component of food packaging, strict compliance with safety regulations applicable to food packaging materials is non-negotiable. The safety risks predominantly originate from potentially toxic substances employed during the fabrication of food freshness indicators. These potentially toxic materials may include certain chemical dyes, heavy metal compounds, or untested synthetic polymers. For instance, some color-changing dyes used in colorimetric freshness indicators might contain aromatic amines [81,82,83,84,85,86], which are known to be carcinogenic [87,88]. Once these hazardous substances are released from the indicators into the packaging environment and ultimately onto the surfaces of foods, they can introduce serious safety hazards.

For example, fresh foods, as is well known, typically possess a high water content. This characteristic creates a sealed packaging environment with elevated humidity levels. In such conditions, water vapor within the package can spontaneously permeate the polymers used in the construction of the freshness indicators. This diffusion process may facilitate the release or leaching of potentially toxic materials from the indicators. For example, Ran et al. [89] prepared the pH indicator films based on soy protein isolate/bromocresol blue and methyl red and reported that water vapor sorption increased the release of soluble matter (e.g., unbound dyes or plasticizers). This study showed that when films with different indicator contents made contact with moisture, the total soluble matter content ranged from 8.21% to 25.56%, which indicated that water vapor could drive the migration of low-molecular-weight compounds from the polymer matrix. Apart from water, many volatile organic gases generated from fresh food could also permeate into the CFFI, potentially leading to the leaching of CFFI components. The leaching mechanism can be influenced by various factors such as the chemical structure of the polymer matrix in the indicator [90], the solubility of the toxic substances, and the storage duration [42,91].

Therefore, it is of utmost importance to comprehensively determine the release behavior of potentially toxic materials in the indicators under realistic packaging conditions. By precisely understanding this release behavior, appropriate measures can be taken to either select safer materials for indicator fabrication or design protective barriers within the packaging to prevent the migration of potential toxic substances.

To achieve this, a series of meticulously designed experiments are required to simulate various real-world scenarios. These should include different storage temperatures (ranging from refrigeration to room temperature), humidity levels (representing diverse packaging environments), and food types (encompassing high-fat, high-protein, and high-carbohydrate foods).

### 3.2. Stability

For most colorimetric food indicators, the gas-sensitive materials (e.g., dyes, pigments, nanoparticles) are embedded into polymers using various technologies [4,92,93,94,95]. Hence, the stability of indicators depends on the physicochemical properties of gas-sensitive materials and polymers, and interaction between them. The main factors that induce the instability of colorimetric food indicators mainly include the humidity, light, oxygen, microorganism, and so on [25,96,97,98,99,100,101].

#### 3.2.1. Effect of Humidity

As previously stated, the high humidity prevalent within food packaging represents a significant challenge in the development of CFFIs, with the leaching of probes being one of the most pressing issues. The underlying principles governing this leaching phenomenon are complex and multifaceted.

**Figure 4:**
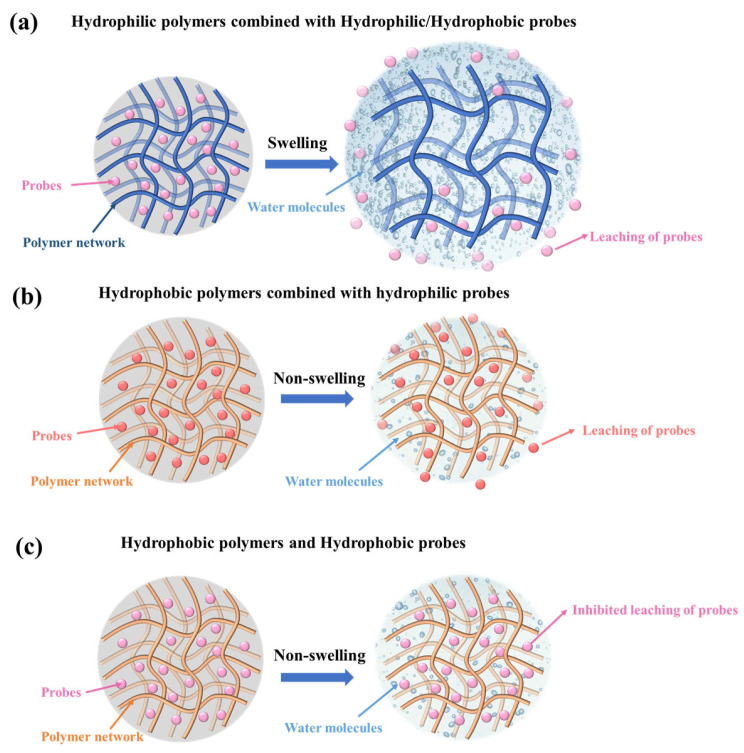
Leakage diagrams of the probes in a high-humidity environment with (**a**) hydrophilic polymers and hydrophilic/hydrophobic probes, (**b**) hydrophobic polymer and hydrophilic probes, and (**c**) hydrophobic polymers and hydrophobic probes.

When considering the nature of the polymers used in the construction of these indicators, their hydrophilic or hydrophobic characteristics play a crucial role. In the case of hydrophilic polymers (e.g., polyvinyl alcohol, carboxymethyl cellulose, and chitosan), it is readily understandable that upon exposure to the high-humidity environment within the package, they will inevitably absorb water vapor [14,33,42]. This absorption leads to swelling of the polymer matrix. As a result, regardless of whether the probes are hydrophilic or hydrophobic, the structural changes in the polymer can facilitate the leaching of probes. For example, as shown in Figure 4a, if the probes are small molecules embedded within the polymer network, the swelling can widen the interstitial spaces, allowing the probes to escape more easily.

Conversely, when dealing with hydrophobic polymers (e.g., polyvinylidene fluoride, polyethylene, and polypropylene) and hydrophilic probes, the leaching behavior of the probes is more intricately linked to the properties of the polymers. As shown in Figure 4b, due to the strong hydrogen bonding forces among water molecules and the inherent repulsive forces between water and hydrophobic polymers, water molecules do not permeate through the film as single entities. Instead, they form water clusters. These clusters then diffuse within the films. If the hydrophobic films do not possess low water permeability coefficients, a significant amount of water clusters will permeate into the films. Once inside, these water clusters can dissolve the hydrophilic probes, thereby triggering the leaching of probes. This process can be further influenced by factors such as the thickness of the polymer film; a thinner film may allow for more rapid water cluster penetration and subsequent probe leaching.

Finally, when both the polymers and the probes are hydrophobic, the leaching problem can be substantially alleviated. Hydrophobic/hydrophobic interactions tend to keep the probes firmly within the polymer matrix (Figure 4c). The absence of a strong driving force for water-induced displacement means that the likelihood of probes being leached out due to humidity-related factors is greatly reduced.

In conclusion, understanding the complex interplay between polymer hydrophilicity/hydrophobicity, probe properties, and the effects of humidity induced water vapor permeation is essential for the successful development of colorimetric food indicators that are resistant to probe leaching, ensuring their reliability and safety for practical applications in the food industry [102].

Many parameters can be used to assess the interaction between CFFIs and water. Among these, the water contact angle (WCA), water vapor permeability (WVP), and water solubility (WS) of the films are commonly utilized [103,104,105,106]. The WCA is defined as the angle formed between a water droplet and the surface of a film, which serves as a critical indicator to evaluate the hydrophobicity or hydrophilicity of the film surface. A high WCA suggests a more hydrophobic surface, while a low WCA indicates hydrophilicity [33,107,108]. Water vapor permeability (WVP) quantifies the rate at which water vapor can pass through the film, which is crucial in understanding how moisture in the environment can interact with the CFFI over time [104,109,110]. Water solubility (WS) determines the amount of film material that can dissolve in water, which may have implications for the integrity of the CFFI and the potential for leaching [111,112]. However, it is important to note that despite their utility in characterizing the CFFI/water interaction, neither WCA, WVP, nor WS can precisely predict the anti-leaching property of the probes within the CFFI.

Currently, the majority of studies aiming to determine the leaching behavior of probes typically involve immersing the CFFI into food simulant solutions, most commonly a water/ethanol mixture [113,114,115]. However, the leaching behavior of CFFI in food simulant solutions cannot be considered equivalent to that in high-humidity environment in all cases. For example, CFFIs constructed from hydrogels exhibit unique properties. Hydrogels possess the ability to absorb water and undergo swelling [116,117]. However, the extent of swelling differs significantly between high-humidity environments and direct immersion in water. In a high-humidity environment, the hydrogel absorbs water vapor gradually, resulting in a relatively lower degree of swelling. In contrast, when immersed in water, the hydrogel is rapidly exposed to a large volume of water, leading to a more pronounced swelling effect [116,118]. For example, Mohammadalinejhad et al. [119] explored the stability of cyanidin-3-glucoside (C3G) loaded alginate hydrogel beads under different relative humidity (RH) conditions. This study investigated the hydrogel beads’ stability at 53% and 97% RH at both room temperature and 4 °C. The experimental results revealed that the hydrogel beads demonstrated high color stability under 97% RH at 4 °C. The color difference, represented by |Δ*E*|, remained ≤3 between day 1 and day 21, indicating minimal color change. Additionally, it was found that the C3G retention rates in the hydrogel beads after 21 days under 97% RH at 4 °C were higher than 64%. This presented the potential of the hydrogel-based CFFI to maintain the integrity of its components, such as probes like C3G, under specific high-humidity storage conditions. Overall, further research is needed to comprehensively understand the leaching behavior of probes from CFFIs under various high-humidity storage conditions.

#### 3.2.2. Effect of Light

**Figure 5:**
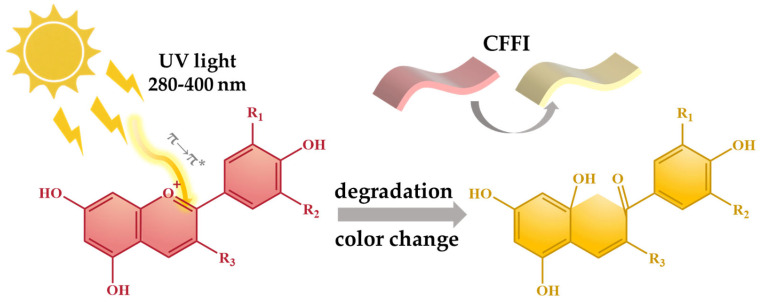
The schematic diagram of anthocyanin degradation under UV light.

Light, especially UV light, also has a great effect on the stability of probes. For example, anthocyanins, which are water-soluble plant pigments widely utilized in CFFIs, are notably vulnerable to the effects of UV light [120,121]. As shown in Figure 5, when anthocyanins are subjected to UV light, the high-energy photons in the UV spectrum are absorbed by the pigment molecules. This absorption process excites the electrons within the anthocyanin structure, causing a shift in their electronic energy levels. As a consequence, the overall electronic structure of the anthocyanin molecules is altered. This change can trigger the cleavage of chemical bonds. In the case of anthocyanins, certain covalent bonds, especially those that are relatively weak due to factors such as resonance effects or steric hindrance within the molecule, are particularly prone to breakage. For example, the C-C double bonds in the anthocyanin chromophore region, which are crucial for its color-bearing properties, can be affected. Once these bonds are broken, the anthocyanin molecule is no longer able to maintain its original structure. Mohammadalinejhad et al. [119] observed that for anthocyanin-containing indicators, the stability of light-exposed samples was inferior to that of their dark-stored counterparts. Under refrigerated conditions, the half-life of anthocyanins in light-exposed samples was approximately one-tenth of that in the same type of samples preserved in the dark. This degradation process often results in a visible color change. The characteristic vibrant hues of anthocyanins, which range from red to purple depending on the pH and the specific anthocyanin variant, may fade or transform into different, often less intense colors. In some cases, significant degradation can lead to a complete loss of pigmentation, rendering the anthocyanin-based CFFI ineffective in accurately signaling food freshness.

#### 3.2.3. Effect of Microorganism

As mentioned above, the proliferation of microorganisms is one of the most significant causes of food spoilage. Since the CFFI is integrated inside the same package as the food, they are also highly likely to be at risk of being invaded by microorganisms. As a result, various extracellular enzymes, such as proteases, lipases, and glycosidases, which are secreted during the metabolic processes of microorganisms, could potentially degrade the key components of the CFFI.

For example, anthocyanins are easily destroyed by oxidases produced by microorganisms, leading to the failure of their color response [122,123]. Additionally, biopolymers such as starch and cellulose, which are used to construct the label structure, will show reduced physical properties and a weakening of mechanical strength due to enzymatic hydrolysis by microorganisms. These changes interfere with the signal transduction mechanism of the indicator label, making it challenging for the label to accurately reflect the true freshness status of the food, thereby misleading consumers’ judgment of food quality.

To effectively address this technical bottleneck, the development of food freshness indicator labels with antibacterial functions has become a research hotspot in the field of food packaging. These indicator labels can inhibit the growth of microorganisms while maintaining the stability and functionality of the label itself [3,124,125,126]. This provides an innovative solution for ensuring food safety and reducing food waste.

#### 3.2.4. Other Factors

In addition to water vapor, several other characteristic gases generated during the deterioration of foods, such as alcohols and acids, can significantly influence the leaching behaviors of substances. For instance, curcumin, a natural pigment with notable antioxidant properties, exhibits near insolubility in water [127]. However, it demonstrates good solubility in ethanol. This solubility difference is attributed to the chemical structure of curcumin, which contains hydrophobic moieties that interact more favorably with the nonpolar regions of ethanol molecules compared to the highly polar water molecules [128]. Zhai et al. [37] conducted a study on the extruded low-density polyethylene/curcumin film. They discovered that when this film was immersed in a water environment (pH buffer solutions), the release rate of curcumin was extremely low. This can be mainly ascribed to the hydrophobic nature of both polyethylene and curcumin. In contrast, Zia et al. [129] investigated the behavior of the polyethylene/curcumin film in an ethanol environment. They found that the film exhibited a high curcumin release rate in ethanol.

Oxygen, a ubiquitous gas in food packaging environments, also plays a critical role in affecting the stability and reactivity of indicator compounds in CFFIs. Many natural pigments, such as anthocyanins, are prone to oxidation reactions when exposed to oxygen. The double bonds in their chemical structures are susceptible to attack by oxygen molecules, leading to structural degradation and changes in their chromogenic properties. For example, Yang et al. [130] motioned that molecular oxygen induced the degradation of anthocyanins via dual mechanisms: direct oxidative reactions and the oxidation of intermediate substances (such as phenolic compounds and metal ions). These oxidized intermediates subsequently underwent chemical interactions with anthocyanins, leading to the formation of colorless or brown degradation byproducts.

Apart from the environmental factors mentioned above that significantly impact the stability of CFFIs, certain factors during the CFFI fabrication process cannot be overlooked under specific circumstances. For example, chitosan is widely used in the development of CFFIs due to its hydrophilicity and excellent film-forming properties [131,132,133,134]. It is well known that chitosan must be dissolved in an acid solution, with acetic acid solution being a common choice. During the preparation of a chitosan-based CFFI, when chitosan is dissolved in acetic acid, not all of the acetic acid may bind with chitosan. If these uncombined acetic acid molecules have not been removed during CFFI fabrication, such as in the drying process, they will continue to volatilize during the utilization of the CFFI, causing a change in the pH value of the CFFI. Since many probes used in CFFIs are pH sensitive, such a pH alteration can trigger the color change of CFFIs. This color change may not be related to the actual freshness of the food, thereby potentially leading to inaccurate indications of food quality. Hence, it is crucial to investigate the self-stability of CFFIs to prevent the adverse influences of such factors.

### 3.3. Sensitivity and Selectivity

Sensitivity refers to the ability of a sensor or indicator to respond to the detection target. A high sensitivity of CFFIs implies that they can identify these molecules at low concentrations, providing an early warning signal of food freshness deterioration, and allowing consumers and food handlers to take timely action [9,14,42]. Selectivity refers to the capacity to distinguish specific target molecules associated with food spoilage from other interfering substances present in the food matrix or packaging environment. For gas-sensitive CFFIs, their sensitivity and selectivity toward target gases are strongly dependent on its composition and structural characteristics [35].

The compositions of CFFIs have significant effect on their sensitivity and selectivity. On one hand, the permeability of target gases in the CFFI polymers primarily governs the speed of color change, while the reaction between the target gases and probes mainly determines the extent of color change, both of which collectively influence sensitivity. On the other hand, the selectivity of CFFIs was mainly determined by the reaction mechanism between probes and target gases.

The structures of gas-sensitive CFFIs are equally important. To enhance sensitivity, porous structures ranging from macropores to mesopores and micropores can be designed to improve gas diffusion efficiency [14,135,136,137]. Thin-film structures further contribute to the response speed of CFFIs, as thinner films reduce the diffusion distance for gas molecules, facilitating faster interactions with the active materials within the film and consequently shortening the overall response time. Additionally, the architecture of the CFFI, such as a core/shell structure [33,138] and a composite-layer structure [139,140,141,142], can be engineered to isolate the active sensing component from the external environment, protecting it from non-specific interactions and improving selectivity.

These carefully designed compositions and structures work in concert to endow gas-sensitive CFFIs with high-performance sensitivity and selectivity, making them effective tools in food freshness monitoring.

## 4. Promising Solutions

### 4.1. Safety Improvement: Biomaterials-Based Solutions

Over the past decade, pH/gas-responsive-based CFFIs have gained popularity due to their use with stimuli-responsive dyes (i.e., synthetic and natural dyes). However, synthetic dyes are known to be toxic and carcinogenic, rendering them unsuitable for food applications. As a result, researchers are exploring alternative solutions, such as biocompatible natural pigments and polymer and nanomaterial-based composite films, which are suitable for ultrasensitive indicators and associated with various dyes [34,36,143].

The European Food Safety Agency (EFSA) has expressed concerns about food safety when intelligent packaging materials come into direct contact with food, emphasizing the risks of particle migration and the obstacles it presents for practical use of such packaging [25]. The European Union for plastics (No 10/2011) sets an overall migration limit (OML) of ≤10 mg/dm^2^ for food contact surfaces for plastics, PVA, and EVOH. However, no specific migration limits are outlined for biodegradable polymers such as starch, carboxymethylcellulose, Arabic gum, cellulose, guar gum, and pectin [144].

Anthocyanins are non-toxic compounds that offer a range of health-promoting bene-fits, including antioxidant, anti-inflammatory, and anticancer properties [145,146]. They can help prevent heart disease, cancer, diabetes, and cognitive disorders, without any adverse effects on health [147,148,149,150]. Betalains are safe for human consumption, but embryotoxicity studies are not conclusive [149]. Moreover, the migration of betalains pigments from films may negatively impact the performance of CFFIs in food freshness monitoring applications [151]. To decrease migration, a covalent complex or crosslinking of polymers can be formed [152]. A stable covalently bonded betalains/cellulose complex was generated through Fischer esterification, and an active/intelligent film crosslinked with alginate by Ca^2+^ was developed [144]. Anthraquinones, a class of natural colorants, are generally considered safe but are not entirely harmless [153,154]. They can cause pathological effects in vivo at high concentrations (between 0.25 and 4 g/kg of body weight), while having insignificant or mild effects at low concentrations. According to Fotia et al. [155], the colorant alizarin exhibits high in vitro activity against bone tumor cell lines and selective effectiveness against malignant cells. It has a strong affinity for calcified tissue and is antigenotoxic and non-mutagenic. Alizarin is therefore an excellent colorant for food freshness indicators, as it poses no health risks and is considered green and safe compared to synthetic pigments [143,155]. Hence, natural pigments have been used to develop intelligent film to overcome the toxicity issue. A cytotoxicity and hemolysis analysis on the safety of Zhang et al.’s [156] developed active film may prove to be a useful reference for CFFI research in the future.

Overall, CFFIs can pose food safety risks by allowing packaging materials to migrate into food during storage. Research in IFP should standardize the distance between CFFIs and food to ensure safety. Indicators placed in package headspace can prevent this, but undesirable components can still diffuse. Therefore, to evaluate CFFI active pigment migration, toxicological and exposure evaluations are required in the near future. Future work should focus on covalent crosslinking to reduce leaching and establishing unified migration limits for biodegradable polymers. The safety of active/intelligent films using natural pigments requires further research, particularly when nanoparticles are used to enhance the films’ barrier properties, functionality, or mechanical performance [157,158].

### 4.2. Stability Improvement

#### 4.2.1. Chemical Modification

The stability of CFFIs can be enhanced through chemical modification or the introduction of new compounds, such as crosslinking agents, polymer blending, hydrophobic compounds, and nanofillers. Table 3 summarizes the common methods and typical examples for improving the stability of CFFIs through chemical modification, along with their respective advantages and disadvantages.

Acylation is regarded as one of the most effective chemical methods to improve the stability of anthocyanins against oxygen, pH changes, and UV light [159]. To improve the stability of anthocyanin-based CFFIs [160], black rice anthocyanins (BRAs) were first acylated with acetic acid, and then the modified black rice anthocyanins (MBRAs) were incorporated into gellan gum (GG). Compared with BRA, the thermal, photostability, and pH stability of MBRA were significantly enhanced. As a result, the color stability of the GG-MBRA film was significantly improved compared with the GG-BRA film during storage under 4 and 25 °C. This study also indicated that curcumin as a co-pigment could significantly enhance the stability of the MBRA and GG-MBRA film.

Chemical crosslinking technology combines molecular chains to create a stronger 3D network, enhancing the mechanical properties, barrier performance, and stability of food packaging films [161]. Crosslinkers significantly impact the physical, chemical, mechanical, opacity, morphological characteristics, and sensitivity of CFFIs [162,163,164]. These agents can be classified into three main types: physical, chemical, and enzymatic crosslinkers. Research focuses on third monomers for biopolymer film crosslinking, but traditional agents like formaldehyde and glyoxal are toxic, necessitating the search for non-toxic, effective new crosslinking agents. While aldehydes such as glutaraldehyde, glyoxal, and formaldehyde are good crosslinking agents, their usage in materials that come into contact with food should be avoided because of the possibility of residue migration [162]. Predominantly, crosslinking agents like dialdehyde starch, metal ions, tannic acid (TA), citric acid (CA), and aldehydes are commonly used in the polymer matrix to prevent pigment degradation and enhance the color stability and sensitivity of CFFIs [25].

Covalent crosslinking between the film-forming polymers of CFFIs can improve their water resistance by forming a denser internal structure and reducing the number of hydrophilic groups available to bind with water. Additionally, the formation of anthocyanins and metal ions improve the stability of anthocyanins and pH-responsive discoloration of the films [165]. Zhao et al. [166] developed a pH-responsive color indicator film based on bovine bone gelatin (BG), chitosan (CS), and blueberry anthocyanin (BA). In this study, CA was used as a safe crosslinking agent, facilitating the crosslinking of BG and CS through polycondensation reactions and ionic crosslinking to form a 3D network structure in the film matrix. The BA-Fe^2+^ chelate, as a pH-sensitive indicator, was successfully incorporated into the crosslinked matrix through hydrogen bonds. It shows that the BA releasing rate from the BG/CS/CA/BA composite film was significantly lower than BG/CS/BA composite film, when they were immersed in different food simulants (distilled water, 10%, 50%, and 90% ethanol solutions), primarily because the BG/CS/CA/BA exhibited good water barrier properties and a denser structure, making it difficult for the internal anthocyanin to be exposed. The release rate of BG/CS/CA/BA-Fe^2+^ is slightly lower than that of BGCSCAA, likely due to the high stability of the BA-Fe^2+^, which effectively prevents the oxidation and degradation of BA [166]. Also, covalent crosslinking between probes and polymers could be the most effective chemical method to enhance the stability of CFFIs [167]. Jia et al. [167] developed a ratiometric fluorescent film by covalently immobilizing the fluorescein isothiocyanate (FITC) and protoporphyrin IX (PpIX) onto cellulose acetate (CA). The film demonstrated excellent fluorescence color stability for up to 5 days when stored with wet tissues, which created a circumstance with high humidity. It exhibited red-to-green color transitions when employed for monitoring the freshness of shrimp and crab. In addition, this method efficiently inhibited the common aggregation-caused quenching of FITC and PpIX, thereby improving the fluorescence color stability of the film.

Similarly, the interaction between metal ions and anionic polysaccharides of CFFI-based films forms stable, thermally irreversible 3D networks, improving the mechanical properties of polysaccharide films. This dense structure reduces water molecules bound to the polymer, enhancing its hydrophobic nature. Run et al. [165] investigated the effect of different metal ions (Ca^2+^, Zn^2+^, and Mg^2+^) on the physical properties and the sensitivity of shrimp freshness monitoring of pectin/carboxymethyl cellulose sodium/anthocyanin films. Crosslinking metal cations with anionic polysaccharides improved the mechanical properties of the film and its stability under high humidity. Moreover, the formation of anthocyanin/metal cation/polysaccharide complexes has significantly enhanced the storage stability of anthocyanin.

The integration of nanoparticles into CFFIs for food freshness monitoring enhances stability, resistance to oxidation, UV light, water, mechanical properties, antimicrobial, antioxidant activity, and gas sensitivity. A cobalt metal/organic framework (Co-MOF) was utilized in CFFIs to develop intelligent films with high color stability and ammonia sensitivity for monitoring food freshness [168,169,170,171]. A study by Feng et al. [171] showed that the sodium alginate/Co-MOF film demonstrated high ammonia sensitivity and color stability for 40 days at 25 °C and 75% RH. Similarly, incorporation of graphene oxide in smart films improved properties such as gas barrier, tensile strength, water resistance, and ammonia sensitivity within 2 s, as well as increased Δ*E* value from 54 to 68 [172].

Overall, the chemically modified CFFI-based food packaging film enhances color stability and physicochemical properties. Further research is needed to develop a hybrid CFFI using multiple additives for improved sensitivity and color stability, such as multiple crosslinkers, two or more color-sensitive agents or co-pigmentation, and different nanofillers.

**Table 3:** Common methods and typical examples for improving the stability of CFFI by chemical modifications.

Types of Modification	Technique	Compounds	Principle	Advantages	Disadvantages	Ref.
Chemical Modification	Acetylate	Black rice anthocyanins (BRA), gellan gum (GG)	Changing the chemical structure of BRA using acetic acid	Enhance the thermal, photostability, and pH stability of anthocyanins	Acylation process is relatively complex; may slightly alter color response range	[160]
	Covalent crosslinking (polymer-polymer) and metal ion chelation	citric acid (CA), bovine bone gelatin, chitosan, anthocyanin-Fe^2+^ chelate	CA facilitates the crosslinking of BG and CS through polycondensation; BA-Fe^2+^ chelate incorporates into the crosslinked matrix through hydrogen bonds	Enhance the thermal stability and water barrier property	May reduce pH sensitivity slightly due to crosslinking density; metal ions may interfere with some color reactions	[166]
	Covalent crosslinking (probe-polymer)	fluorescein isothiocyanate (FITC), protoporphyrin IX (PpIX), cellulose acetate (CA)	Covalently immobilize the FITC as indicator and PpIX as internal reference onto CA, respectively	Enhance stability, inhibit aggregation-caused quenching of probes	Require specific reaction conditions (e.g., precise control of pH, temperature, reaction time)	[167]
	Metal Ion Crosslinking	Metal ions (Ca^2+^, Zn^2+^, and Mg^2+^), pectin, carboxymethyl cellulose sodium, anthocyanin	Crosslinking of metal cations with anionic polysaccharides; Metal ions chelate with anthocyanins	Enhance stability of the film under high humidity and the storage stability of anthocyanins	Safety concern regarding whether metal ions will migrate into food	[165]
	Nanofiller incorporation	Nano cobalt metal/organic framework (Co-MOF), sodium alginate (SA)	Blending Co-MOF with SA matrix	Enhance long-term storage color stability	MOF synthesis is costly; may increase production complexity	[171]

#### 4.2.2. Physical Modification

The physical modification can enhance color stability and other physicochemical properties of CFFIs. Advanced preparation techniques, such as encapsulation, electrospinning, and 3D printing, offer unique advantages, including minimal active compound concentration, uniform thickness, low cost, and ease of production, while also allowing for varying production costs at an industrial scale. Table 4 summarizes the common methods and typical examples for improving the stability of CFFIs through physical modification, along with their respective advantages and disadvantages.

Fascinatingly, the encapsulation technique protects CFFIs from environmental influences, enhancing their bioavailability and slow delivery while also preventing degradation due to heat, light, and oxygen [25]. Nanoencapsulation enhances surface areas, quantum size effects, mechanical, and pH-responsive indicator film barrier properties, resulting in improved outcomes [173]. Particularly, metal/organic frameworks (MOFs) have been utilized for pigment encapsulation due to their finely adjustable porosity, broad surface areas, and surface functionalities. Zeolitic imidazolate framework-8 (ZIF-8) is a highly stable and minimally cytotoxic type of MOF among various types. For instance, Zhang et al. [39] reported a photothermally stable phytochemical dye, alizarin, which has been conjugated with ZIF-8 (AL@ZIF-8), enhancing color stability under visible and UV light. The nanoparticles show significant color changes in various pH environments, demonstrating potential for meat freshness monitoring. Oktay et al. [174] successfully encapsulated anthocyanins by utilizing the intermolecular hydrogen bonds between the nitrogen atom of 2-methylimidazole in the ZIF-8 MOF and the phenolic hydroxyl groups of anthocyanins.

Moreover, emulsion encapsulation technology enhances the stability of materials susceptible to CFFI, ensuring high encapsulation efficiency of active indicator compounds in CFFIs. This technique ensures color stability, controlled release, biocompatibility, and improved pH/gas sensitivity, making it a cost-effective method for developing smart CFFIs. Recently, researchers have developed an emulsion encapsulation technique to improve the stability of a sensitive material, thereby increasing the volatility and hydrophobicity of CFFIs and acting as an active compound for shelf-life enhancement [175,176,177,178]. Wang et al. [176] found that gelatin/alizarin/lavender essential oil Pickering emulsion films have high color stability and the color changed from yellow to red and eventually purple red when the pH increased from 2 to 11. Despite repeated exposure to ammonia and acetic acid vapor, these films exhibited a distinctive reversible color shift, showing significant sensitivity to ammonia with a color shift occurring in just 2 min. Hashim et al. [175] created a hybrid film using agar, methylcellulose, Chinese purple cabbage, sunflower wax, and Origanum compactum essential oil (as a carrier of active compound) based on CFFIs for chicken breast monitoring. The oil significantly improved film hydrophobicity, color stability, and ammonia sensitivity, with an exposure time of 40 min [175]. Additionally, the encapsulation technique, despite potential challenges, improves the stability, physicochemical properties, and functional characteristics of CFFIs by reducing pH/gas sensitivity compared to the unencapsulated natural pigments. Therefore, the development of CFFIs requires careful consideration of the optimal concentration of the active compound and emulsion incorporation.

Numerous studies have employed layer-by-layer deposition to create multilayer CFFIs for food freshness monitoring [174,179,180]. Researchers have developed a new strategy to create leaching-resistant CFFIs by enhancing the hydrophobicity of the active component through layer-by-layer assembly during preparation, making it suitable for fresh foods with high humidity levels [14,179]. Shi et al. [181] developed a hydrophobic sodium alginate/anthocyanin/cellulose nanocrystal-based CFFI by using nano silica (NS) as a waterproofing layer. They optimized the concentrations and formation methods of the NS layer (spraying (S), coating (C), and impregnation (I)). Out of the three, NSI-based CFFIs have a high color stability and distinctive color change corresponding to fish spoilage during 14 days of storage [181]. Yong et al. [182] developed high-quality antibacterial and antioxidant bilayer films using curcumin, SIP, and chitosan, exhibiting high pH sensitivity at pH 8 for beef preservation and freshness monitoring. Zhao et al. [183] prepared a composite film using natural dye, chitosan, trehalose, and PVA through the electrospinning technique. The film was modified with polyethylene terephthalate (PET) film, creating a “sandwich” structure. This resulted in a “sandwich” pH indicator, which showed superior efficacy and stability in monitoring pork freshness [183]. Intriguingly, the 3D printing method offers distinct advantages over other label preparation methods. Three-dimensional printing is a digital technique that creates objects based on digital models, offering advantages such as faster production, lower cost, free design of label shapes, size, and porosity, avoiding errors, greater flexibility in product design, less material waste, and excellent color sensitivity [184,185]. The 3D printing approach can be used for CFFI fabrication, enabling the creation of smart/intelligent packaging by combining distinct materials in different layers with unique functionality. Tang et al. [184] used curcumin in 3D printing to create pH-sensitive indicator films with high stability for grass carp freshness monitoring using curcumin/oregano oil Pickering emulsion/potato starch/polyvinyl alcohol ink.

Overall, the physical modification method has been employed to attain high color stability, enhancing the other physicochemical and functional properties.

**Table 4:** Common methods and typical examples for improving the stability of CFFI by physical modifications.

Types of Modification	Technique	Compounds	Principle	Advantages	Disadvantages	Ref.
Physical Modification	MOF encapsulation	Alizarin, zeolitic imidazolate framework-8 (ZIF-8), polyvinyl alcohol (PVA), sodium alginate (SA)	Conjugation of alizarin with ZIF-8 (AL@ZIF-8), then blending with PVA/alginate	Enhance the stability under visible and UV light	Encapsulation efficiency may vary; ZIF-8 may limit mass transfer of target gases	[39]
	Emulsion encapsulation	Gelatin, alizarin, lavender essential oil (LEO)	LEO was emulsified into LEO Pickering emulsions (LEOPs); LEOPs and alizarin were then integrated into the 3D network gelatin matrix	Enhance the color stability	Require strict control of optimal concentration of active compound and emulsion incorporation	[176]
	Layer-by-layer deposition	SA, anthocyanins, cellulose nanocrystal, nano silica (NS)	Impregnation of NS as waterproof layer	Enhance hydrophobicity and color stability	Additional layers may increase film thickness, potentially slowing gas diffusion	[181]
	Electrospinning	Blueberry anthocyanin, petunia dye, chitosan, trehalose, PVA	Electrospinning to form nanofiber films with “sandwich” structure (PET film coating)	Enhance color uniformity and stability	High equipment cost; challenges in large-scale production	[183]
	3D printing	Curcumin, oregano oil Pickering emulsion, potato starch (PS), PVA	Using curcumin/oregano oil Pickering emulsion/PS/PVA based gel as ink, the indicator film was developed by 3D printing technology	Enhance water resistance and stability	Printing parameters (e.g., nozzle size) require precise optimization	[184]

### 4.3. Sensitivity and Selectivity Improvement

Numerous advanced techniques, such as encapsulation, crosslinking, 3D printing, and electrospinning can modify sensitive compounds in CFFIs, thereby enhancing sensitivity and other barrier properties.

#### 4.3.1. Porous Structures

Porous structures enhance the performance of CFFIs in intelligent packaging by improving sensitivity, selectivity, and responsiveness to pH changes or gas emissions from food spoilage. Their high surface area, tunable pore size, and excellent adsorption properties enable faster interaction between indicator dyes and target analytes, resulting in more vivid, rapid, and reliable color changes, improving real-time freshness monitoring. MOF-based CFFIs have led to significant breakthroughs in the visual detection of food freshness because of their remarkable sensitivity, simple visual effects, easy operation, and affordability. A novel class of crystalline porous materials known as MOFs are distinguished by their hole-like structures, which are created when metal ions or clusters establish coordination connections with organic ligands [186,187]. Recently, MOF-based CFFI research work (88%) has been deeply studied in the past three years for food freshness monitoring [188]. MOF-based CFFIs are being developed for monitoring food freshness, with high ammonia sensitivity but no pH sensitivity, while natural pigment-encapsulated MOFs have both [39,169,170,171,189,190]. Khan et al. [191] developed a multifunctional film using gelatin/carrageenan and red cabbage extracts incorporating Cu-MOFs for smart food packaging, enhancing pH, ammonia sensitivity, UV-blocking, barrier, and antibacterial properties. Gomes et al. [192] found that adding plasticizer glycerol (20–30 wt%) to a pyranoflavylium/cellulose acetate matrix affects pH sensitivity. Glycerol-free films do not change color, while 20 wt% Gly showed a good color change between pH 6 and 7, with a Δ*E* value ranging from 37 to 75. Glycerol-containing films’ porosity and increased water permeability enhance pH sensitivity [192]. Furthermore, volatile compounds (TVB-N, ammonia, histamine, methylamine, etc.) can improve gas sensitivity in CFFIs by diffusing within cellulose-based films’ 3D porous network, reacting with natural pigment molecules. The composition material porous structure impacts pH/gas sensitivity, making biopolymer and co-material selection crucial for high sensitivity in CFFIs to monitor food freshness.

#### 4.3.2. Electrospinning

Electrospinning is a versatile method for producing ultrafine nanofibers, with significant potential for large-scale production of CCFIs due to its pore size and high surface area. This method improves film sensitivity by exposing the indicator material to its full potential, resulting in polymeric nanofiber films with high encapsulation of active compounds, flexibility, porosity, and functionality for improving food freshness monitoring. These distinctive characteristics make it possible to meet the needs of colorimetric detection, including consistent color change, high sensitivity, and fast detection time. Whereas electrospinning can enhance the sensitivity and color stability of indicator films by increasing the number of active sites [193], nanofiber-based CFFIs also improve sensitivity in response to acidic and alkaline conditions, and exhibit increased interaction sites between the immobilized active compound in the CFFI and total volatile basic nitrogen (TVB-N) from meat spoilage [194]. Weng et al. [106] compared casting and electrospinning methods for the development of CFFIs based on intelligent packaging film preparation, finding nanofiber films exhibited better volatile ammonia response sensitivity and correlated well with the deterioration of pork, as indicated by the thiobarbituric acid reactive substance (TBARS) value, which reflects the oxidation degree of unsaturated fatty acids in meat, due to their hydrophilicity and porous structure. Zhao et al. [183] made a pH indicator for monitoring pork freshness using blueberry anthocyanin and petunia dye. The indicator was embedded in nanofiber films, chitosan, trehalose, and polyvinyl alcohol. The “sandwich” structure ensured even dye distribution and environmental stability. It was highly responsive to ammonia vapor, with a detection range covering critical freshness thresholds of pork [183]. The stability of pH-sensitive indicator films can be improved by incorporating a hydrophobic film-forming matrix and a protective layer. However, this may reduce sensitivity and monitoring accuracy. The extended gas diffusion pathway from the protective layer also decreases sensitivity. To solve this issue, Cetinkaya et al. [195] suggested processing hydrophobic film-forming materials by electrospinning to develop indicator films, which have greater porosity and pH sensitivity. Therefore, incorporation of hydrophobic materials or bilayer nanofiber-based CFFIs can improve film stability, producing a denser structure and stable color changes. Electrospinning technology faces challenges due to high equipment requirements and costs, hindering control and commercialization in the food indicator industry. The composite morphology can be regulated, and various materials can be used for functional products. The process is scalable but requires specialized equipment for fiber alignment, and potential issues include nozzle clogging, bead formation, non-uniform diameter, and fiber quality. For instance, essential oil, a volatile material, can be better encapsulated using the coaxial electrospinning method, which offers better encapsulation and more controllable volatile material retention compared to uniaxial nozzle electrospinning. Electrospinning can enhance sensitivity, but stability is only slightly improved. Likewise, further research is needed to optimize/develop the electrospinning model and new hybrid CFFI with high encapsulation or uniform coating of the CFFI’s active compound, improving color stability and sensitivity of naturally sourced pH-sensitive indicator films for large-scale production.

#### 4.3.3. Three-Dimensional Printing

Conventional printing technologies like inkjet, gravure, and screen printing have drawbacks like expensive printers, ink cartridges, tools, long pre-press processes, high-level waste, technical limitations, and high cost-per-piece for small orders. These technologies also require expensive tools and additional equipment, such as gravure plates [185]. A trend is emerging where 3D printing is used to create indicators, sensors, and electronic tags. Consumer-level additive manufacturing is divided into stereolithography and extrusion-based 3D printing. These 3D-printed devices offer alternatives to conventionally fabricated devices for monitoring food quality, package integrity, and food authentication. Three-dimensional printing, a new additive manufacturing technique, is being used to customize CFFI labels. This technique, which constructs the final model layer by layer through molten filament extrusion, can enhance design flexibility, reduce manufacturing costs, and minimize sample-to-sample errors. Although the solution casting approach has many drawbacks, it requires extra drying procedures and leads to significant shrinkage of the films or labels. For creative food-intelligent packaging, 3D printing provides an alternative to conventional casting techniques, addressing concerns related to sample size and the management of colorimetric indicator material content in CFFIs to achieve high sensitivity. Like fused deposition modelling (FDM), 3D printing is gaining popularity due to its potential advantages [196]. IFP is currently unaffordable for the food industry due to higher product costs. Research focuses on developing cost-saving strategies, with 3D printing being a strong contender. This method can fabricate sensors, indicators, and tags for intelligent food packaging, which could be integrated into regular food packaging. The main issue with anthocyanins in high-humidity food packaging is their leaching into the environment, which disrupts sensor performance by causing anthocyanin migration from the interior to the surface [42]. To overcome this, Zhai et al. [42] developed an anthocyanins-encapsulated bigel that was extruded in a spiral shape onto a polyvinylidene fluoride (PVDF) film using 3D printing, resulting in a composite film with excellent anti-leaching ability. To prevent the leaching of anthocyanins, the researchers developed a hydrogel-in-oleogel bigel. The PVDF-bigel film showed a red-to-light purple color change when used to monitor beef and salmon freshness in a customized packaging device. Remarkably, the anthocyanin-encapsulated bigel was a cost-effective and robust solution for large-scale industrial production due to its easy, cost-effective, and robust fabrication process [42]. Tang et al. [184] utilized curcumin in 3D printing technology to create pH-sensitive indicator films (curcumin/oregano oil Pickering emulsion (COPE)/potato starch (PS)/polyvinyl alcohol (PVA) based gel as ink) for monitoring freshness changes in grass carp storage. For the development of sustainable 3D printing inks for use in biotechnology, water treatment, food and agriculture, energy, and bioplastics, and a variety of biomaterials, including proteins, polysaccharides, and other polymers, are utilized. To enhance printability and expedite development, it is recommended to explore existing 3D printing methods derived from natural sources, along with AI-assisted techniques [197]. According to Grira et al. [197], AI-assisted enhancement techniques are crucial in predicting material properties, selecting smart materials, and optimizing printing parameters in the development of 3D bioprinting materials. AI technology can soon improve the accuracy of 3D printing for CFFIs, allowing for easy optimization to enhance their sensitivity and productivity. Limited research has utilized 3D printing for developing IPF for food freshness monitoring, but it is an evolving technique that can enhance RH stability, reduce pigment concentration usage, and improve pH/gas sensitivity. Further research is needed to develop novel CFFIs using nanofillers, mixed sensitive pigments, and composite materials using 3D/4D printing for high sensitivity.

#### 4.3.4. Composition Optimization

Film composition and microstructure are among the other factors that influence the sensitivity of CFFI-based intelligent packaging. Typically, protein and polysaccharide composite films are used as solid supports in CFFIs due to their water resistance, mechanical and barrier properties, compatibility with halochromic dyes, and low production costs. Composite films made from multiple constituents offer superior physicochemical properties and functions, prepared with biopolymers, synthetic polymers, or a combination of both [198]. Also, it offers superior physicochemical properties and functions compared to single polymer films. Biopolymers (i.e., starch and chitosan) are blended with synthetic polymers PVA to create biodegradable composites for halochromic dye immobilization. These composites have stronger tensile strength than starch or chitosan films but are less suitable for freshness indicators due to acetic acid. Based on the CFFI’s active compound sources of origin, content, co-pigment, and concentration may impact its pH/gas sensitivity. For example, Kan et al. [199] conducted a study on anthocyanin composition in 14 plant extracts, finding that starch/PVA (SP) haskap berry (HBE) and SP Chinese bayberry (CBE) had the deepest colors and highest Δ*E* values due to their high total anthocyanin contents. The acylated anthocyanin compositions showed similar pH sensitivity [199]. Acylated anthocyanins demonstrate better color stability in aqueous environments than non-acylated ones. However, their use in intelligent food packaging can be challenging due to the potential for subtle color shifts that may not be noticeable to the naked eye [25]. Moreover, the addition of co-pigmentation to CFFIs can improve pH and ammonia sensitivity. Bao et al. [200] found no significant difference in Δ*E* value when high concentration chondroitin sulfate (>3 mg/mL) was added to potato starch/blueberry anthocyanins film. However, the addition of chondroitin sulfate increased pH sensitivity from 17.35 to 19.42 for pH 2 to 12 and ammonia sensitivity (Δ*E* value) from 6.02 to 9.13 [200].

Since the color change of CFFIs is mostly caused by the influence of the sensitive pigment, even a little amount of nanofiller added to composite films has no discernible effect. According to Zheng et al. [172], films with varying cellulose nanocrystal concentrations exhibited minor color variations, which were attributed to structural changes in anthocyanin under different pH levels. Contractually, the CMC/anthocyanin film’s color becomes shallow due to dissolution and anthocyanin leaching into water. However, by precisely tuning the content of graphene oxide (GO), the resultant smart films display enhanced water resistance and gas barrier and tensile strength. The CMC/anthocyanin/GO_2_ (2:0.02:0.0066, g/g/g) film has high sensitivity to volatile ammonia, increasing its Δ*E* value from 54 to 68 within 2 s [172].

Overall, sensitivity and selectivity are improved by optimizing material structures and compositions. Porous designs (e.g., MOFs, nanofibers via electrospinning) increase surface area for faster analyte interaction, while 3D printing enables customizable architectures for targeted gas diffusion. Compositional adjustments (e.g., adding co-pigments, graphene oxide) further enhance response accuracy. Despite progress, challenges include reducing cross-reactivity with non-target gases and scaling up complex fabrication processes for industrial use.

### 4.4. Commercial Value

CFFI-based smart packaging is a promising new technology that is rapidly growing in the food packaging sector. However, due to high costs and limited integration into existing packaging, these systems are still often used [201]. Currently, only a few colorimetric indicators are used in commercial packaging, primarily to indicate food quality and safety. For example, freshness indicators like RipeSense™, SensorQ™, and FreshTag™ are representative of this type [4]. For a variety of reasons, the commercialization of these CFFI products is still a way off. Because of their versatility, the kind of indicator pigment, and the method of manufacture, food freshness indicators are continually being developed. The durability of the dye during usage may be impacted by the compatibility of the natural pigments with the biopolymer matrix. Challenges may also arise from the compatibility between the biopolymer matrix and the indicator, which could affect the organoleptic properties of foods and lead to issues with food safety and quality. The migration from the packaging material into the food may potentially hinder the commercialization of food packaging based on biopolymer freshness indicators and natural pigments. Additionally, the majority of the current generation of intelligent food packaging based on CFFIs is created in research laboratories without taking production costs into account. While CFFI-based intelligent sensors should be less than 10% of the whole cost of packaging, advanced smart materials make up 50% to 100% of the total cost [25,201]. Food freshness monitoring is hindered by the high costs and complex methods required to prepare CFFIs, necessitating the search for more affordable raw materials and the optimization of the preparation process to reduce production costs [10]. To lower the cost of materials and processing, increase the economic viability of intelligent packaging, and investigate a greater variety of high-value food items, additional research is required. The depletion of natural raw material supplies might result from the growing market demand for biopolymers and natural materials. Plant materials may be expensive for commercial use, are reliant on the weather, and are only accessible in small amounts. By shortening the plant development cycle under controlled conditions and increasing raw material output and availability without compromising the natural source, plant tissue culture technology can be used to overcome these challenges.

A further hurdle in the development of intelligent packaging is the optimization of existing technologies to enhance user-friendliness. Customers seeking to buy food obtain product information via social media or other mobile platforms, with 84% of consumers indicating a preference for using their smartphones over examining product labels during transactions [202]. Moreover, the global population, comprising 1–8.8% of men and 0.4–3% of women, is significantly affected by color vision deficiency. To overcome this, smart packaging systems using wireless technology, connected to cloud servers or databases via smartphones and portable devices, can initiate colorimetric sensors for complex information interpretation [25]. Recently, fewer studies have used smartphones to monitor ammonia sensitivity and real-time food spoilage monitoring as colorimetric smart packaging labels. A smartphone app called Smart Food was developed for quantitative food deterioration studies using fish gelatin (FG)/red cabbage anthocyanin/carbon dot (CD) films for chicken spoilage monitoring, using UV irradiation and carbon dots as crosslinkers. The color response of the UV-treated FG films containing 100 mg/l (FG-UV-CD100) was measured, showing high ammonia sensitivity [203]. Additionally, further research indicates that integrating colorimetric indicators into WeChat mini-programs and smartphone apps offers a promising technology for fast detection of food quality in 5–30 s, including meat, Atlantic salmon, and oysters [201,204]. More research is needed to integrate smartphones with CFFI-based intelligent food packaging films for freshness monitoring at every stage of the supply chain. Hence, colorimetric sensing technology can only benefit society if a careful balance is struck between social responsibility, environmental stewardship, and scientific growth.

In conclusion, CFFIs hold significant commercial potential in the food industry, particularly for food quality and safety monitoring. However, its widespread commercialization is hindered by multiple challenges, including high production costs, compatibility issues between natural pigments and biopolymer matrices, potential migration concerns, and limited consideration of mass production feasibility in current research. Additionally, optimizing user-friendliness, such as integrating with smartphone technologies to cater to consumer preferences and address color vision deficiency, is crucial for broader adoption. Future research should focus on reducing costs through affordable raw materials and process optimization, enhancing material compatibility and safety, leveraging plant tissue culture to ensure raw material supply, and advancing smartphone integration for seamless information access across the supply chain. Striking a balance between scientific innovation, environmental sustainability, and social responsibility will be key to unlocking the full potential of CFFI-based smart packaging.

## 5. Conclusions

CFFIs have become a key technology in intelligent food packaging, enabling real-time visual monitoring of perishable food quality through color changes. This review synthesizes its progress, limitations, and solutions based on the existing literature. CFFIs have developed into various forms like colorimetric films and sensor arrays, with applications in meats, seafoods, fruits, and vegetables. For meats and seafoods, they target compounds such as TVB-N and H_2_S using materials like pH-sensitive dyes and porphyrins, while for fruits and vegetables, they focus on CO_2_, ethylene, and pH changes, showing potential in reducing food waste. However, three core challenges persist: safety concerns from toxic leaching of synthetic dyes and polymers, with high humidity accelerating migration; stability issues due to humidity, UV light, and microbial enzymes affecting performance; and sensitivity and selectivity gaps, with single-component films struggling with low-concentration analytes and cross-reactivity. Promising solutions supported by research include using natural pigments and biopolymers for safety, chemical modification and physical techniques for stability, and porous structures, electrospinning, 3D printing, and composition optimization for sensitivity. Commercialization is hindered by high costs and accessibility issues, so future research should focus on hybrid materials, AI-driven optimization, and smartphone integration. Overall, the CFFI’s potential is evident, but addressing these challenges with evidence-based solutions is crucial for transforming sustainable food systems.

## Data Availability

No new data were created or analyzed in this study. Data sharing is not applicable to this article.

## Abbreviations

CFFI	Colorimetric food freshness indicators
IFP	Intelligent food packaging
TTI	Time–temperature indicators
RFID	Radio-frequency identification tags
WCA	Water contact angle
WVP	Water vapor permeability
WS	Water solubility
